# Exploring the Effects of Players’ Numbers and Court Size on Tactical-Technical Performance Analysis of Novice Players in Basketball Small-Sided Games

**DOI:** 10.5114/jhk/190400

**Published:** 2024-12-19

**Authors:** Walber Jose Figueiredo de Souza, Filipe Manuel Clemente, Samuel da Silva Aguiar, Larissa Pittner, Erivaldo Machado Araújo, Matheus de Souza Rocha, Francielli Evelin Lopes Silva, Ana Filipa Silva, Henrique de Oliveira Castro

**Affiliations:** 1 Physical Education Department, Federal University of Mato Grosso, Cuiabá, Brazil.; 2 Escola Superior Desporto e Lazer, Instituto Politécnico de Viana do Castelo, Viana do Castelo, Portugal.; 3 Research Center in Sports Performance, Recreation, Innovation and Technology - SPRINT, Melgaço, Portugal.; 4 Department of Biomechanics and Sport Engineering, Gdansk University of Physical Education and Sport, Gdansk, Poland.; 5 Physical Education Department, University Center UDF, Brasília, Brazil.

**Keywords:** task constraints, team sports, tactics, constrained games, technique

## Abstract

This study aims to examine the tactical behavior, decision-making, and technical skills of young novice basketball players in small-sided games (SSGs) with different numerical configurations and court sizes. Participants were 16 novice male players aged between 11 and 15 years with no competitive experience. A total of 13 games were played, comprising nine SSG formats with numerical equality, superiority, and inferiority, in two court sizes: a full court (FC) and a half court (HC). In SSGs played in the FC, pass efficacy was significantly higher (p < 0.05) in 5 vs. 4 and 4 vs. 3 formats, while dribble efficacy was significantly higher (p < 0.05) in the 2 vs. 1 HC format. The 3 vs. 3 FC format showed greater (p < 0.05) shot efficacy. Reception efficacy was significantly higher (p < 0.05) in the 2 vs. 1 HC format, as well as rebound efficacy. However, appropriate passes were significantly lower (p < 0.05) in the 4 vs. 3 FC format. Dribble efficacy was significantly greater (p < 0.05) in the 2 vs. 1 HC format and appropriate shots were significantly greater (p < 0.05) in the 2 vs. 1 HC format. Regarding defensive and offensive technical-tactical actions, the 3 vs. 3 HC format presented significantly higher values (p < 0.05) of support, while ball marking was significantly greater (p < 0.05) in the 3 vs. 2 HC format. In conclusion, this study indicates that smaller (balanced and unbalanced) SSG formats tend to enhance the frequency, effectiveness, and appropriateness of attacking and defensive behaviors, particularly those involving direct actions.

## Introduction

Small-sided games (SSGs) are characterized by changes in the functional structures in the game, such as court size, the number of players, rules, scoring, limitations of actions, tactical strategies, rest intervals, and a training regime (Castro et al., 2022; Clemente et al., 2021a). SSGs allow the development of the physical, technical, and tactical aspects in an integrated way, due to the characteristics of the game format (Sansone et al., 2020). This aspect can be particularly interesting as it aligns with the physical, technical, and tactical demands inherent in the game (Pérez-Chao et al., 2023; Pérez-Ifrán et al., 2022). In addition, the limitations of players and space allow more offensive and defensive actions to occur in relation to the formal game, being more intense and allowing the players to have more contact with the ball (Clemente, 2016; Klusemann et al., 2012). In this sense, SSGs are presented as a methodological alternative for the development of sports performance (Davids et al., 2013).

Considering the Newell's constraints approach (Newell, 1986), it is anticipated that the design of SSGs, along with the simultaneous application of various task constraints, can influence players' behaviors as they engage with the challenges presented in the exercise. This, in turn, is expected to promote impacts on the technical execution and tactical behavior of the players (Timmerman et al., 2017). Consequently, manipulating elements such as the format of play (e.g., the number of players involved and their numerical relationships) and pitch configuration (e.g., pitch dimensions) can subsequently influence how players respond to the dynamic nature of the match, ultimately constraining the technical execution (Kostrna, 2022).

In terms of technical performance, smaller SSG formats show higher frequencies of technical actions in invasion sports in general (Clemente et al., 2021a). Specifically in basketball, more dribbling and shooting actions were observed in the 3 vs. 3 game format in male semi-professional athletes (Sansone et al., 2020), more short-distance shooting in the 2 vs. 2 game format with elite male players (Klusemann et al., 2012), higher frequency of dribbling and shooting in the 2 vs. 2 game format with U14 athletes (Arslan et al., 2022), more technical actions per minute in smaller game formats, and higher frequency of defensive actions in the national competition level for U14 compared to U16 (Clemente et al., 2020).

In terms of tactical performance, studies with SSGs in basketball show learning of tactical principles regarding the occupation of spaces in numerically unbalanced formats with male U13 players (Poureghbali et al., 2020), and higher frequency of space creation with and without the ball in the 3 vs. 3 format on the half court with youth male athletes at the national and regional levels (Bredt et al., 2018). In addition, a study by Bredt et al. (2023) compared the offensive and defensive tactical behavior in various SSG formats with equality and numerical superiority and rule changes in U14 and U15 male athletes participating in national and regional competitions. Results showed that SSGs with numerical superiority enabled greater offensive performance (Bredt et al., 2023).

Práxedes et al. (2021) showed that the effect of the game-based approach presents a greater transfer of tactical knowledge to the real game in high school physical education students with no previous experience in basketball. Regarding decision-making, Diniz et al. (2022) used SSGs to analyze decision-making of tactical-technical actions in male and female schoolchildren with no previous experience in basketball and found that SSGs with fewer players and numerical unbalancing favor the understanding of the logic of the game (3 vs. 2) in the learning phase. Tallir et al. (2012) compared the 3 vs. 3 and 5 vs. 5 game formats in youth athletes (11 and 12 years old) and found that the 3 vs. 3 format allowed to improve decision-making and motor skills.

Despite the growing number of studies with SSGs in basketball, there are only few studies that have analyzed tactical behavior and decision-making in young players with competitive experience (Bredt et al., 2023) or without any previous experience in the sport (Diniz et al., 2022). Thus, it is necessary to analyze the tactical-technical and decision-making variables of youth athletes and different game formats (court size and the number of players). In this sense, this study aimed to evaluate tactical behavior, decision-making, and technical skills of youth basketball players in SSGs with numerical equality and superiority/inferiority using two court sizes. We hypothesized that half-court games would allow more frequent tactical actions compared to full-court games. Additionally, we expected more effective technical skill actions in games with numerical superiority/inferiority.

## Methods

### Study Design

This study employed a descriptive cross-sectional study design to examine and compare the tactical and technical performance of basketball players in various game formats and court sizes. The research was conducted in a single day, during a specific stage of the season. All the games were played over one day, and players had a 10-min rest interval before each small-sided game. The games lasted from 2 to 5 min, following recommendations by Clemente et al. (2021b). The games were conducted under specific temperature and environmental conditions (27°C). Furthermore, the game sessions were all held in the morning, to ensure uniformity across formats.

### Participants

The study utilized a convenience sampling strategy, recruiting a specific basketball team to participate. From the available players, 16 novice male players between the ages of 11 and 15 years were selected, with a mean age of 12.75 ± 1.25 years and mean training experience of 1.53 ± 0.33 years. Players were not federated and had no competitive experience at a regional, national or international level. A demographic data questionnaire was applied to characterize the sample. The total sample size used in this study (n = 16) conferred a statistical power of 95% (β = 0.95) with a significance level of 5% (α = 0.05) and a large effect size (*d* = 0.8). Participants in this study were classified at tier 2 of the Participants Classification Framework, indicating their competitive level within the sport (McKay et al., 2022). These players were regularly engaged in two weekly training sessions, each lasting 1 h and 30 min. To be eligible for the study, participants were required to meet the following criteria: (i) to take part in all prescribed playing formats; (ii) not presenting any injuries or illnesses before and during the experiment; and (iii) to be part of the team before the study's commencement. All ethical procedures were respected. The study was conducted following the principles of the Declaration of Helsinki, and approved by the Ethics Committee of the Universidade Federal de Mato Grosso (protocol code: 5.916.260; approval date: 28 February 2023). Before their participation, athletes and their legal guardians were thoroughly briefed on the study's design, potential risks, and benefits. Subsequently, the legal guardians provided their informed consent for the athletes' involvement in the study, and both the guardians and players signed the required documentation.

### Measures

The Game Performance Assessment Instrument (GPAI) was used for the analysis. The GPAI is an instrument used to assess game performance through technical, tactical, and decision-making development in game situations (Oslin et al., 1998). The GPAI allows analyzing both situations with the ball (offensive) and without the ball (defensive and offensive) in different game situations. In addition, the analysis is carried out through game observation, either in real-time or based on recordings (Memmert and Harvey, 2008). The GPAI has been used for performance evaluations in team sports such as soccer (Fortes et al., 2018) and basketball (Diniz et al., 2022; Leonardi et al., 2019; Wright et al., 2005). For the present study, the GPAI items used for the analyses were tactical and technical as well as decision-making components (Memmert and Harvey, 2008).

Tactical components analyzed were coverage (provides appropriate defensive coverage, serving as a backup for a player involved in a direct action against a player with the ball), guarding/marking (appropriate guarding/marking of an opponent who may or may not have the ball), and supporting (provides appropriate support for a teammate with the ball by being in a position to receive a pass (proper return of the performer to a recovery position between skill attempts)). The absolute frequency of actions was recorded for each of these components (Diniz et al., 2022; Memmert and Harvey, 2008).

Technical components considered included efficient execution of passing, dribbling, shooting, catching, and rebounding. These actions were considered “effective” if successful, while “ineffective” when unsuccessful (Diniz et al., 2022).

Decision-making was analyzed in actions with the ball (dribbling, passing, and shooting). For this, each action in the game was classified as appropriate (refers to an adequate decision based on the specific game situation) or inappropriate (refers to a mistaken decision based on the specific game situation). However, only decision-making was analyzed for each specific situation, and the course of the play was not considered (Diniz et al., 2022).

### Procedures

SSGs took place in a tournament format. Thus, players were divided into two teams balanced in the tactical-technical-physical aspects by the head coach. Both teams were composed of eight players each and were named “Team A” and “Team B”. Participants were identified by numbers from 1 to 16. Participants received a numbered vest equivalent to their identification to facilitate visualization during evaluation. In addition, each team wore different colored vests.

During the games, the head coach and evaluators were not allowed to provide players with tactical or technical instructions, only verbal encouragement. As a way of motivating the players, medals were offered to the team who scored the most points at the end of the experiment (Klusemann et al., 2012). The following score was adopted for all game formats: 3 points for the winning team; 2 points in the event of a tie; and 1 point for the losing team. Data collection took place during an extra training session.

In total, 13 games were played with nine SSG formats with numerical equality, superiority, and inferiority, in two different court sizes (a full and a half court). Games in the full court (FC) playing area (28 x 15 m) were: numerical equality (5 vs. 5, 3 vs. 3, 4 vs. 4), superiority and inferiority (4 vs. 3, 5 vs. 4). Games in the half court (HC) playing area (15 x 14 m) were: numerical equality (3 vs. 3, 2 vs. 2), superiority and inferiority (3 vs. 2, 2 vs. 1). Players were evaluated individually by two experts, in a total of 76 individual observations.

**Figure 1:**
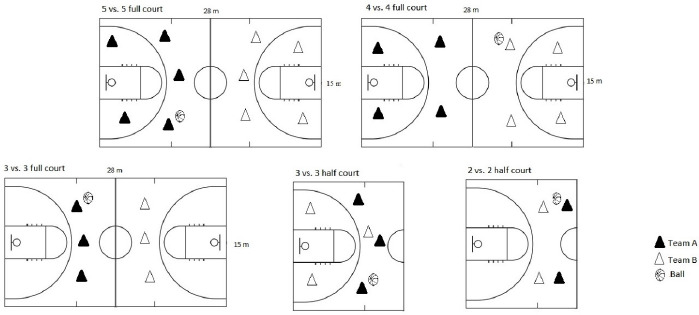
SSG formats with numerical equality.

**Figure 2:**
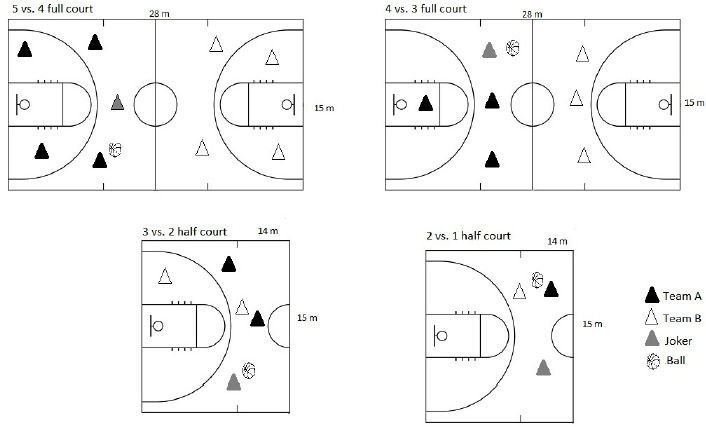
SSG formats with numerical superiority/inferiority.

The sequence of games took place alternately so that participants could have a passive rest between games. Initially, there were seven games with numerical equality (in this sequence: 5 vs. 5 FC, 3 vs. 3 HC, 2 vs. 2 HC, 3 vs. 3 FC, and 4 vs. 4 FC) and then six games with numerical superiority/inferiority (in this sequence: 2 vs. 1 HC, 3 vs. 2 HC, 4 vs. 3 FC, and 5 vs. 4 FC). SSGs with numerical equality were applied to five (Figure 1), while those with numerical superiority/inferiority to four game formats (Figure 2).

Game duration ranged from 2 to 5 min: 5 vs. 5 FC lasted 5 min; 4 vs. 4 FC, 4 vs. 3 FC, and 5 vs. 4 FC lasted 4 min; 3 vs. 2 HC, 3 vs. 3 HC, and 3 vs. 3 FC lasted 3 min; 2 vs. 2 HC and 2 vs. 1 HC lasted 2 min. All players participated in all game formats, not considering the games they participated in as jokers, which were chosen randomly at the time of the game, by the head coach.

### Statistical Analysis

Data normality and homogeneity were assessed using Shapiro-Wilk and Levene's tests, respectively. To evaluate the reliability within and between observers, the intraclass correlation coefficient (ICC) was calculated using all data from the SSGs. The results were presented as medians and interquartile ranges and compared using the Friedman's test with Dunn's as a post hoc test. The *r* effect size was calculated and classified as small (0.10), medium (0.30), or large (0.50) (Fritz et al., 2012). Statistical significance was defined as *p* < 0.05. All procedures were carried out using GraphPad Prism (v. 6.0), G*Power (v. 3.1), and Statistical Package for the Social Sciences (v. 21.0 for Windows, SPSS Inc., Chicago, IL, USA).

## Results

Intra and inter observers’ reliability showed acceptable ICC values (ICC = 0.948 [IC 95% = 0.941_0.954]; F_(932,932)_ = 37,631; *p* < 0.0001).

**Figure 3:**
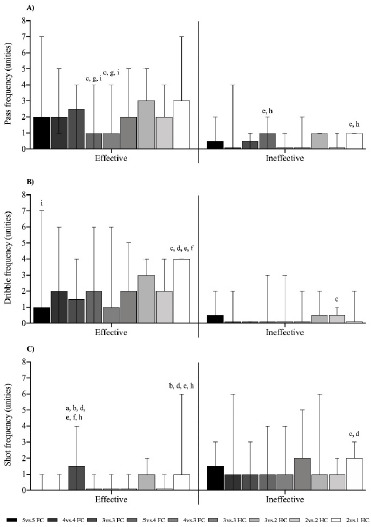
Efficacy of the technical-tactical actions of passing, dribbling and shooting. a, b, c, d, e, f, h: between-games statistical difference (p < 0.05) from the 5 vs. 5 FC, 4 vs. 4 FC, 3 vs. 3 FC, 5 vs. 4 FC, 4 vs. 3 FC, 3 vs. 3 HC, 3 vs. 2 HC, 2 vs. 2 HC, and 2 vs. 1 HC format, respectively

Figure 3 shows the efficacy of the technical-tactical actions of passing, dribbling, and shooting analyzed using the GPAI. Pass efficacy was significantly higher (*p* < 0.05) in 5 vs. 4 FC and 4 vs. 3 FC SSGs compared to 3 vs. 3 FC, 3 vs. 2 HC, and 2 vs. 1 HC SSGs, while pass inefficacy was significantly higher (*p* < 0.05) in 5 vs. 4 FC and 2 vs. 1 HC SSGs compared to 4 vs. 3 HC and 2 vs. 1 HC SSGs (Figure 3A). Dribble efficacy was significantly higher (*p* < 0.05) in the 2 vs. 1 HC compared to the 3 vs. 3 FC, 5 vs. 4 FC, 4 vs. 3 FC, and 3 vs. 3 HC formats, whereas dribble inefficacy was higher (*p* < 0.05) in the 2 vs. 2 HC format compared to the 3 vs. 3 FC format (Figure 3B). The 3 vs. 3 FC format showed greater (*p* < 0.05) shot efficacy compared to the 5 vs. 5 FC, 4 vs. 4 FC, 5 vs. 4 FC, 4 vs. 3 FC, 3 vs. 3 HC and 2 vs. 2 HC formats. The 2 vs. 1 HC format also showed higher shot efficacy (*p* < 0.05) compared to the 4 vs. 4 FC, 5 vs. 4 FC, 4 vs. 3 FC, and 2 vs. 2 HC formats. Shot inefficacy was higher (*p* < 0.05) in the 2 vs. 1 HC format in comparison to the 3 vs. 3 FC and 5 vs. 4 FC formats (Figure 3C).

**Figure 4:**
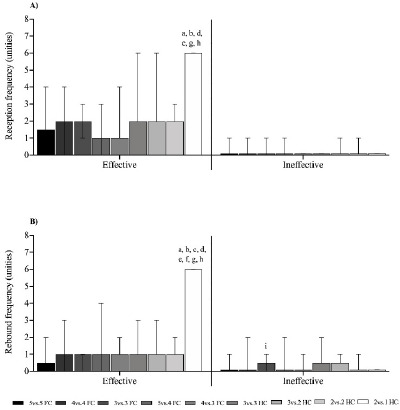
Efficacy of the technical-tactical actions of reception and rebound. a, b, c, d, e, f, h: between-games statistical difference (p < 0.05) from the 5 vs. 5 FC, 4 vs. 4 FC, 3 vs. 3 FC, 5 vs. 4 FC, 4 vs. 3 FC, 3 vs. 3 HC, 3 vs. 2 HC, 2 vs. 2 HC, and 2 vs. 1 HC format, respectively

Figure 4 shows the efficacy of the technical-tactical actions of reception and rebound analyzed using the GPAI. Reception efficacy was significantly higher (*p* < 0.05) in the 2 vs. 1 HC format compared to the 5 vs. 5 FC, 4 vs. 4 FC, 5 vs. 4 FC, 4 vs. 3 FC, 3 vs. 2 HC, and 2 vs. 2 HC formats. No differences (*p* > 0.05) were found in reception inefficacy (Figure 4A). Rebound efficacy was significantly higher (*p* < 0.05) in the 2 vs. 1 HC compared to all other game formats, while rebound inefficacy was higher (*p* < 0.05) in the 3 vs. 3 FC compared to the 2 vs. 1 HC format (Figure 4B).

**Figure 5:**
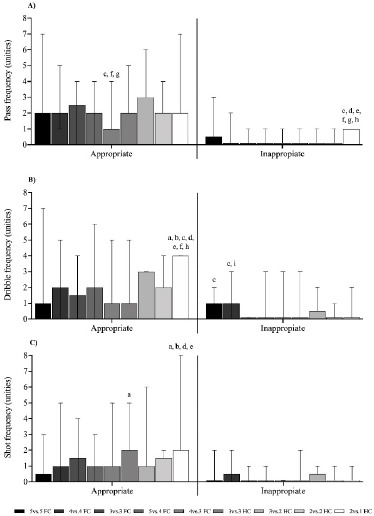
Medians of the pass, dribble, and shot related to the decision-making. a, b, c, d, e, f, h: between-games statistical difference (p < 0.05) from the 5 vs. 5 FC, 4 vs. 4 FC, 3 vs. 3 FC, 5 vs. 4 FC, 4 vs. 3 FC, 3 vs. 3 HC, 3 vs. 2 HC, 2 vs. 2 HC, and 2 vs. 1 HC format, respectively

Figure 5 shows the medians of the pass, dribble, and shot related to the decision-making component of the GPAI. Effective passes were significantly lower (*p* < 0.05) in the 4 vs. 3 FC format compared to the 3 vs. 3 FC, 3 vs. 3 HC, and 3 vs. 2 HC formats, whereas ineffective passes were higher (*p* < 0.05) compared to the 3 vs. 3 FC, 5 vs. 4 FC, 4 vs. 3 FC, 3 vs. 3 HC, 3 vs. 2 HC, and 2 vs. 2 HC formats (Figure 5A). Dribble effectiveness was significantly higher (*p* < 0.05) in the 2 vs. 1 HC compared to all other game formats. On the other hand, ineffective dribbles were higher in 5 vs. 5 FC and 4 vs. 4 FC SSGs (*p* < 0.05) compared to the 3 vs. 3 FC format. Additionally, the 4 vs. 4 FC format showed higher (*p* < 0.05) dribble ineffectiveness compared to the 2 vs. 1 HC format (Figure 5B). Effective shots were significantly greater (*p* < 0.05) in the 2 vs. 1 HC compared to 5 vs. 5 FC, 4 vs. 4 FC, 5 vs. 4 FC, and 4 vs. 3 FC formats. Effective shots were also higher (*p* < 0.05) in the 3 vs. 3 HC compared to the 5 vs. 5 FC format. There were no significant differences (*p* > 0.05) in ineffective shots between different game formats (Figure 5C).

**Figure 6:**
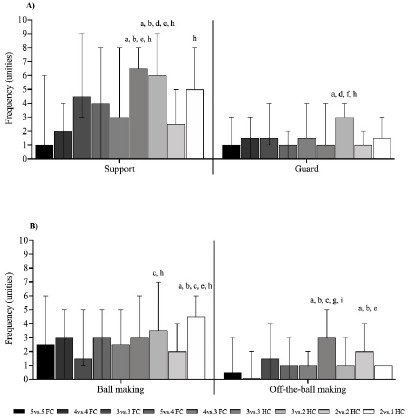
Defensive and offensive technical-tactical actions with and without the ball. a, b, c, d, e, f, h: between-games statistical difference (p < 0.05) from the 5 vs. 5 FC, 4 vs. 4 FC, 3 vs. 3 FC, 5 vs. 4 FC, 4 vs. 3 FC, 3 vs. 3 HC, 3 vs. 2 HC, 2 vs. 2 HC, and 2 vs. 1 HC format, respectively

Figure 6 shows the defensive and offensive technical-tactical actions with and without the ball included in the GPAI. The 3 vs. 3 HC format presented significantly higher support values (*p* < 0.05) compared to the 5 vs. 5 FC, 4 vs. 4 FC, 4 vs. 3 FC, and 2 vs. 2 HC formats. Similarly, the 3 vs. 2 HC format showed higher support values (*p* < 0.05) compared to the 5 vs. 5 FC, 4 vs. 4 FC, 5 vs. 4 FC, 4 vs. 3. FC, and 2 vs. 2 HC formats. The 2 vs. 1 HC format showed higher support values (*p* < 0.05) in comparison to the 2 vs. 2 HC format (Figure 6A). There were also significantly higher guarding values (*p* < 0.05) in the 3 vs. 2 HC format compared to the 5 vs. 5 FC, 5 vs. 4 FC, 3 vs. 3 HC, and 2 vs. 2 HC formats (Figure 6A). Ball marking was significantly higher (*p* < 0.05) in the 3 vs. 2 HC format compared to 3 vs. 3 FC and 2 vs. 2 HC formats. Similarly, the 2 vs. 1 HC format showed higher (*p* < 0.05) ball marking than 5 vs. 5 FC, 4 vs. 4 FC, 3 vs. 3 FC, 4 vs. 3 FC, and 2 vs. 2 HC formats (Figure 6B). Off-the-ball marking was significantly greater (*p* < 0.05) in the 3 vs. 3 HC compared to the 5 vs. 5 FC, 4 vs. 4 FC, 4 vs. 3 FC, 3 vs. 2 HC, and 2 vs. 1 HC formats. Similarly, off-the-ball marking was higher in the 2 vs. 2 HC (*p* < 0.05) compared to 5 vs. 5 FC, 4 vs. 4 FC, and 4 vs. 3 FC formats (Figure 6B).

## Discussion

The aim of this study was to perform a comprehensive analysis of the strategic conduct, decision-making processes, and technical proficiencies exhibited by youth basketball players during different SSGs. Specifically, SSGs with numerical equality and numerical superiority were played in half-court or full-court settings. The underlying hypothesis stated that distinct patterns would emerge based on these variables. Specifically, we anticipated a higher frequency of tactical actions performed during HC games in comparison to FC games. Moreover, we hypothesized a more proficient technical skill execution in games with numerical superiority.

The research findings elucidate several notable trends encompassing varied formats of basketball's SSGs. Noteworthy observations emerge when analyzing larger setups, such as 4 vs. 3 or 5 vs. 4 configurations, in conjunction with imbalanced scenarios. Such settings are discernibly linked to an augmented occurrence of both effective and ineffective passes. This finding contradicts a prior original study, which indicated that concerning offensive maneuvers, a rise in the number of opponents resulted in a decrease in passing, driving, and controlled actions. Conversely, an increase in the number of teammates was linked to a greater amount of time spent in attacking situations (Torrents et al., 2016).

On the contrary, the 2 vs. 1 scenario within a half-court setting distinctly exhibited an increase in both successful and unsuccessful shot attempts, adept dribbling, effective receptions, and rebounds. These findings are consistent with a previous study (Clemente et al., 2021b) demonstrating that smaller formats typically elevate the counts of shots, receptions, and rebounds when compared to larger formats. In the realm of appropriateness evaluation, the 2 vs. 1 format within the HC setting stood out for its heightened propensity towards suitable dribbling and shot-taking frequencies. However, it was equally marked by a notably elevated frequency of misguided passes. A contrasting observation came to light in the context of the 4 vs. 3 format, which exhibited a significantly amplified rate of pass execution.

Shifting the focus towards defensive dynamics, the study underscores a distinct proclivity for ball marking in the 2 vs. 1 and 3 vs. 2 formats within the HC context. Meanwhile, support actions manifested themselves with remarkable prominence in the 3 vs. 3, 3 vs. 2, and 2 vs. 1 formats within the HC. Off-the-ball marking gained salience in the 3 vs. 3 and 2 vs. 2 formats within the HC, while guarding tendencies were pronounced in the 3 vs. 2 format within the HC.

Prior research has demonstrated that smaller basketball SSGs, such as 2 vs. 2 setups, typically result in approximately 40% more technical executions compared to larger formats like 4 vs. 4 (Klusemann et al., 2012). These findings are in line with the notion that reducing the number of players results in increased engagement and involvement of each participant. However, it is worth noting that interactions involving numerical equality or imbalance can yield distinct outcomes. For instance, numerical imbalance may generate more open spaces off the ball, potentially influencing the observed patterns differently (Bredt et al., 2022).

As indicated in our study, the 2 vs. 1 HC game emerged as a game setting of particular interest. This format significantly increased the occurrence of both successful and unsuccessful shot attempts, and conducted to an increase in dribbling skills, efficient receptions, and rebounds. The presence of two attacking players against one defender creates a significant advantage, as the defender is compelled to close in on the player with the ball, thereby reducing the opportunity to cover the passing lane for the opponent who is providing an option to his teammate. Moreover, it indicated a trend for more proficient dribbling and shot-taking frequencies, while also facilitating increased support to teammates on offense. On the defensive front, this arrangement exhibited a clear inclination for ball marking. Remarkably, this game format revealed the most significant disparities compared to the other game formats investigated.

These findings are in line with the notion that smaller game formats tend to generate a higher frequency of actions (Clemente et al., 2020), and when coupled with numerical imbalances, can lead to variations in available spaces and opportunities (Bredt et al., 2022). Particularly in the context of the 2 vs. 1 format, complex tactical behaviors such as unified attacking are somewhat diminished (Castelão et al., 2014). The only defender in this game is focused primarily on ball marking, rather than off-the-ball actions. The primary objective of the defender is trying to steal the ball from the attacking player in possession, while maintaining an organized defensive structure to limit opportunities for the attacking player without the ball, creating a potential disruption in the opponent's passing lines.

The presence of a single player in the defensive role drives the two attacking players to strategize on overcoming this defense. This can involve attempting direct dribbles to bypass the defender or creating opportunities for passing and shooting. The isolated nature of the defender allows for more space behind this defender, thereby increasing the perception of risk-taking in individual duels. Consequently, this justifies the heightened efficacy and frequency of dribbling and shot-taking actions. Additionally, the increased reception efficiency can be attributed to the absence of a direct defender marking the player without ball possession, reducing the pressure under this action.

In essence, the 2 vs. 1 scenario presents a distinctive tactical and technical dynamic, where the single defender's vulnerability to the attacker’s strategies creates increased opportunities for dribbling and shot attempts. Furthermore, the augmented space behind the defender contributes for offensive players to increase the probability of taking actions with higher risk, enhancing the overall tactical interactions in this SSG format.

Conversely, larger SSG formats present a more challenging environment for engaging in one-on-one encounters, such as dribbling. This is especially pronounced in balanced formats, where the available space without ball possession is significantly reduced (Bredt et al., 2022). In larger formats, there is an increased emphasis on attack for providing support to the player in possession of the ball and employing passing strategies to disrupt the opponent's positioning. This strategic approach aims to create opportunities for penetration or shot attempts. This phenomenon is substantiated by prior research, which indicated that larger setups such as a 4 vs. 3 format favour ball circulation to generate off-the-ball spaces (Padilha et al., 2107). Notably, our study brought to light an intriguing observation regarding such setups: the imbalanced condition yielded a more advantageous environment for successful and appropriate passes. Evidently, numerical superiority appears to exert a notable influence on enhancing pass efficacy, potentially due to the facilitation of locating a teammate without the immediate pressure of direct opponent marking (Diniz et al., 2022). Furthermore, the occurrence of the 5 vs. 4 and 4 vs. 3 formats in our study, both taking place in the FC, offers additional insights. This setting serves to reinforce the notion that a larger playing area provides a more favorable environment for attackers to benefit from defensive imbalances between opponents. The intelligent use of ball circulation, through well-timed passes, emerges as a pivotal strategy. This approach facilitates the identification of opportune moments to exploit unguarded spaces or to execute shots, thus overlapping the defensive actions performed by the opposing team.

Despite the insights presented in our study, it is important to acknowledge its limitations, which may warrant caution when drawing broad generalizations. One notable limitation lies in the relatively small participant sample (n = 16), which may restrict the scope of extrapolation to a wider population. Furthermore, it is prudent to interpret the conclusions within the context of the players' age and experience level (average playing experience of approximately 1.25 years). Another notable limitation is the absence of repeated SSG matches. Given the context-dependent nature of SSGs, their outcomes can be influenced by a multitude of factors, contributing to considerable within-player variability (Clemente et al., 2022). This underscores the potential for results to be influenced by the specific match context encountered. In light of these limitations, while our findings offer valuable insights into the realm of basketball SSGs, they should be interpreted with consideration, emphasizing the need for cautious extrapolation to broader contexts.

In future research, several aspects can be explored to enhance the robustness and depth of understanding regarding SSGs in basketball. Firstly, expanding the participant sample size would contribute to a more comprehensive and representative analysis. To provide a more comprehensive assessment, conducting repeated matches for each game format is advisable. This approach would enable to analyze variations in performance and outcomes, while also accounting for potential fluctuations inherent in dynamic game scenarios. Incorporating additional layers of complexity, such as tactical metrics based on players' positions is another possible aspect to be explored in the future. By delving into the spatial dynamics and positional interactions within the game, a richer contextual understanding can be gained. This could shed light on the underlying mechanisms of the observed events and outcomes during SSGs, offering a more comprehensive interpretation.

In terms of practical implications, especially within the context of novice players, the findings of this study offer valuable insights for optimizing training strategies. Specifically, the utilization of smaller formats, such as the 2 vs. 1 configuration within the HC, emerges as a promising option. This format proves favorable to sharpening essential skills like dribbling, shooting, and individual defensive actions. On the other hand, the study indicates that larger formats conducted in a FC setting provide athletes with a different tactical dimension. These configurations allow to refine passing strategies and enhance off-ball defensive actions. By strategically integrating these formats into training sessions, coaches and practitioners can adjust their approaches to foster versatile skill development and game understanding among novice players.

## Conclusions

Given the hypotheses that half-court games would lead to more frequent tactical actions compared to full-court games and that games with numerical superiority/inferiority would contribute to more effective technical skill actions, the study's results unveiled notable patterns. Specifically, larger formats, such as 4 vs. 3 and 5 vs. 4, along with imbalanced formats, were associated with significantly more effective and ineffective passes. In contrast, the 2 vs. 1 HC format demonstrated a significant increase in effective and ineffective shot frequency, dribbling, effective reception, and rebounds.

Analyzing appropriateness, the 2 vs. 1 HC format exhibited significantly higher appropriate dribble and shot frequencies, although it also displayed significantly more inappropriate passes. Conversely, the 4 vs. 3 format demonstrated a significantly higher pass frequency.

Considering defensive behaviors, ball marking was notably higher in the 2 vs. 1 HC and 3 vs. 2 HC formats, while support actions were significantly greater in the 3 vs. 3 HC, 3 vs. 2 HC, and 2 vs. 1 HC formats. Off-the-ball marking was significantly more prevalent in the 3 vs. 3 HC and 2 vs. 2 HC formats, while guarding was significantly higher in the 3 vs. 2 HC format.

In conclusion, this study underscores that smaller (balanced and unbalanced) SSG formats tend to enhance the frequency, effectiveness, and appropriateness of attacking and defensive behaviors, particularly those involving direct actions. Conversely, larger formats of play, even in imbalanced scenarios like 4 vs. 3 or 5 vs. 4, appear to be more suitable for promoting passing actions.

## ORCID iD

Walber Jose Figueiredo de Souza: https://orcid.org/0000-0002-5268-8500

Ana Filipa Silva: https://orcid.org/0000-0002-1772-1272

Samuel da Silva Aguiar: https://orcid.org/0000-0002-9057-4096

Larissa Pittner: https://orcid.org/0000-0001-5223-0682

Erivaldo Machado Araújo: https://orcid.org/0000-0002-5444-4163

Matheus de Souza Rocha: https://orcid.org/0000-0002-2614-9714

Francielli Evelin Lopes Silva: https://orcid.org/0000-0001-6149-6044

Filipe Manuel Clemente: https://orcid.org/0000-0001-9813-2842

Henrique de Oliveira Castro: https://orcid.org/0000-0002-0545-164X
